# Supplementary material for: Immunometabolic determinants of long-term response in leukemia patients receiving CD19 CAR T cell therapy
Source: Nat Commun. 2026 Feb 20;17:2967. doi: 10.1038/s41467-026-69857-4 (PMC13035890; doi:10.1038/s41467-026-69857-4)
Supplement: Supplementary file 1 — Supplementary Information [file 41467_2026_69857_MOESM1_ESM.pdf]

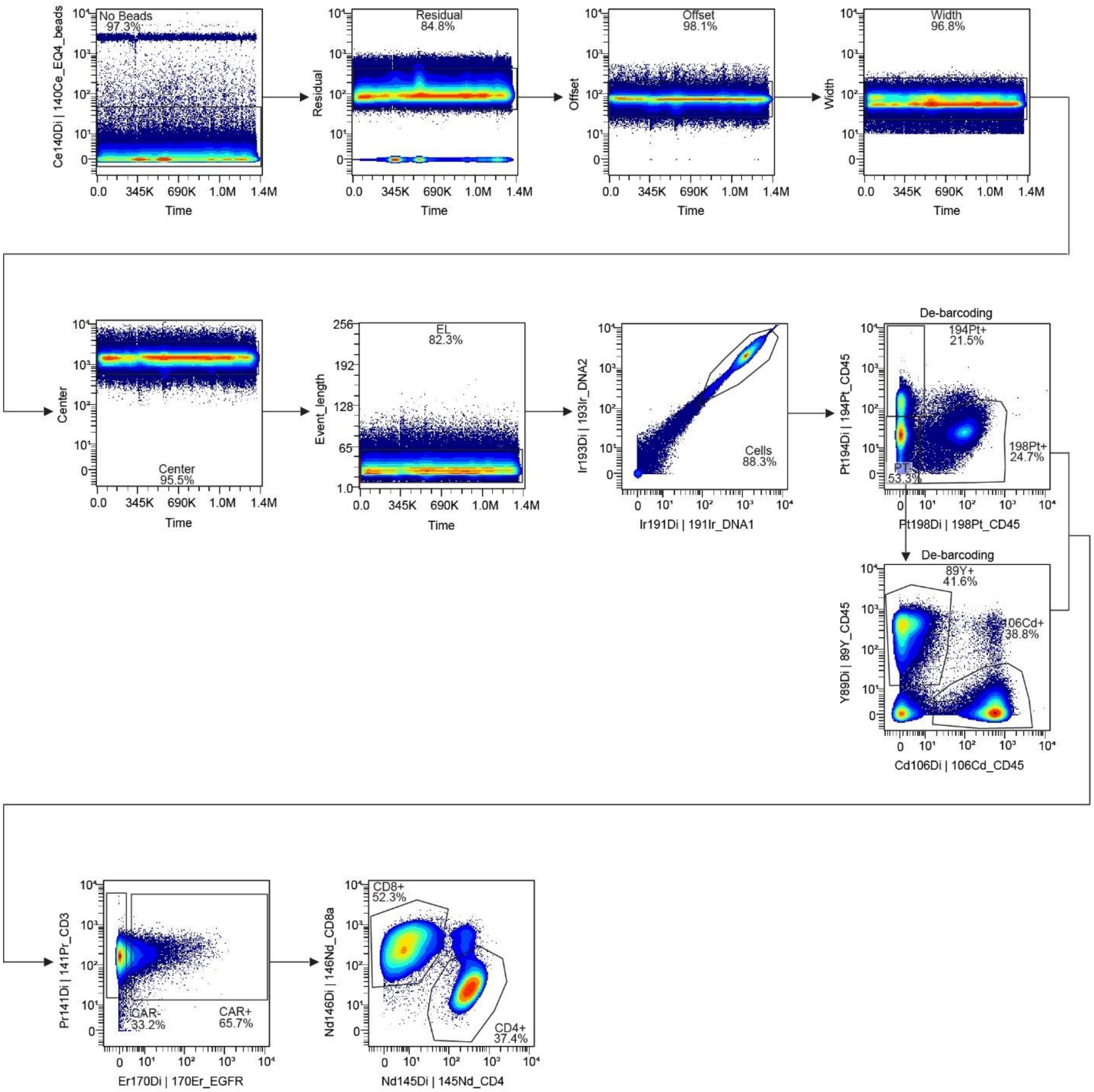

Supplementary Fig. 1: Representative of gating strategy to detect CAR- and CAR+ T cells by mass cytometry. CyTOF data were cleaned using the Gaussian parameter discrimination method, and live cells were detected by excluding beads (140Ce) and manually gating on DNA (191Ir/193Ir) and viability (195Pt). Patients' samples were manually de-barcoded (CD45; 194Pt/198Pt/89Y/106Cd), and CAR T and non-CAR T cells were identified using anti-CD3 (141Di) and anti-huEGFRt (170Er), followed by CD4 (145Nd) and CD8 (146Nd) metal-tagged antibodies. Corresponds to: Fig. 1i-m, Fig. 3b-d and l, Fig. 4d-e and n.

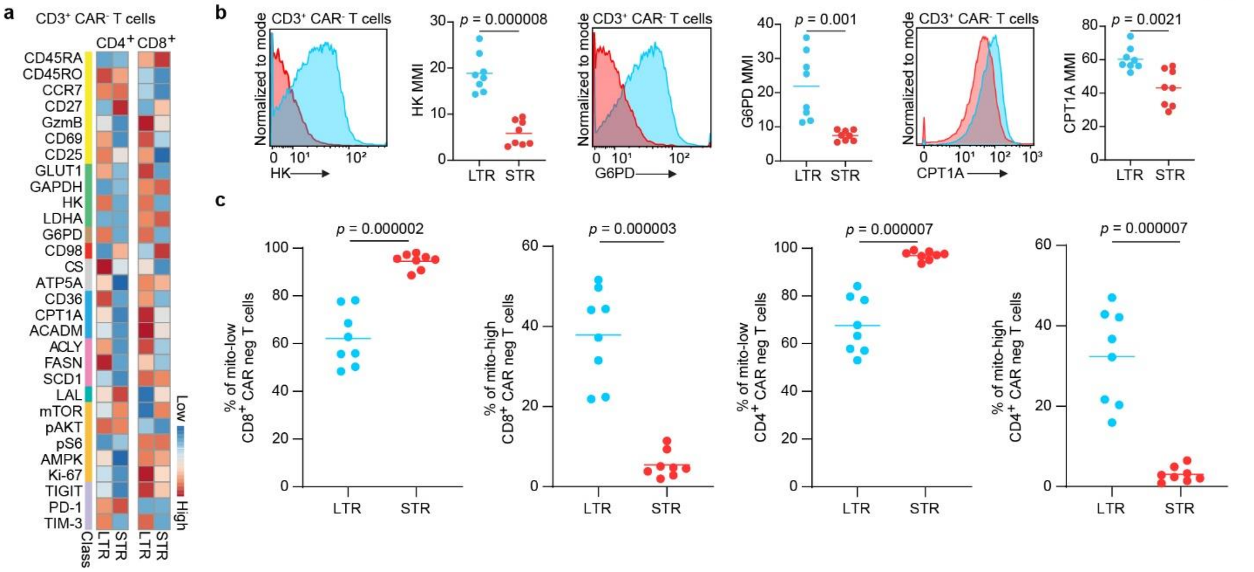

Supplementary Fig. 2: CD3+ CAR- T cells of long-term responders show increased mitochondrial mass and expression of key enzymes involved in oxidative phosphorylation, fatty acid oxidation, and pentose phosphate pathway activity. a Heat map indicating z-score normalized median expression of all CyTOF markers assessed in CD4+ and CD8+ CAR- T cells. **b** Representative histograms and analysis of statistically significant proteins in CD3+ CAR- T cells. **c** Analysis of MitoTracker expression in CD4+ and CD8+ CAR- T cells products. Data are mean from $n = 16$ patients. Significance levels were calculated using two-tailed unpaired Student's *t*-tests. Source data are provided as a Source Data file.

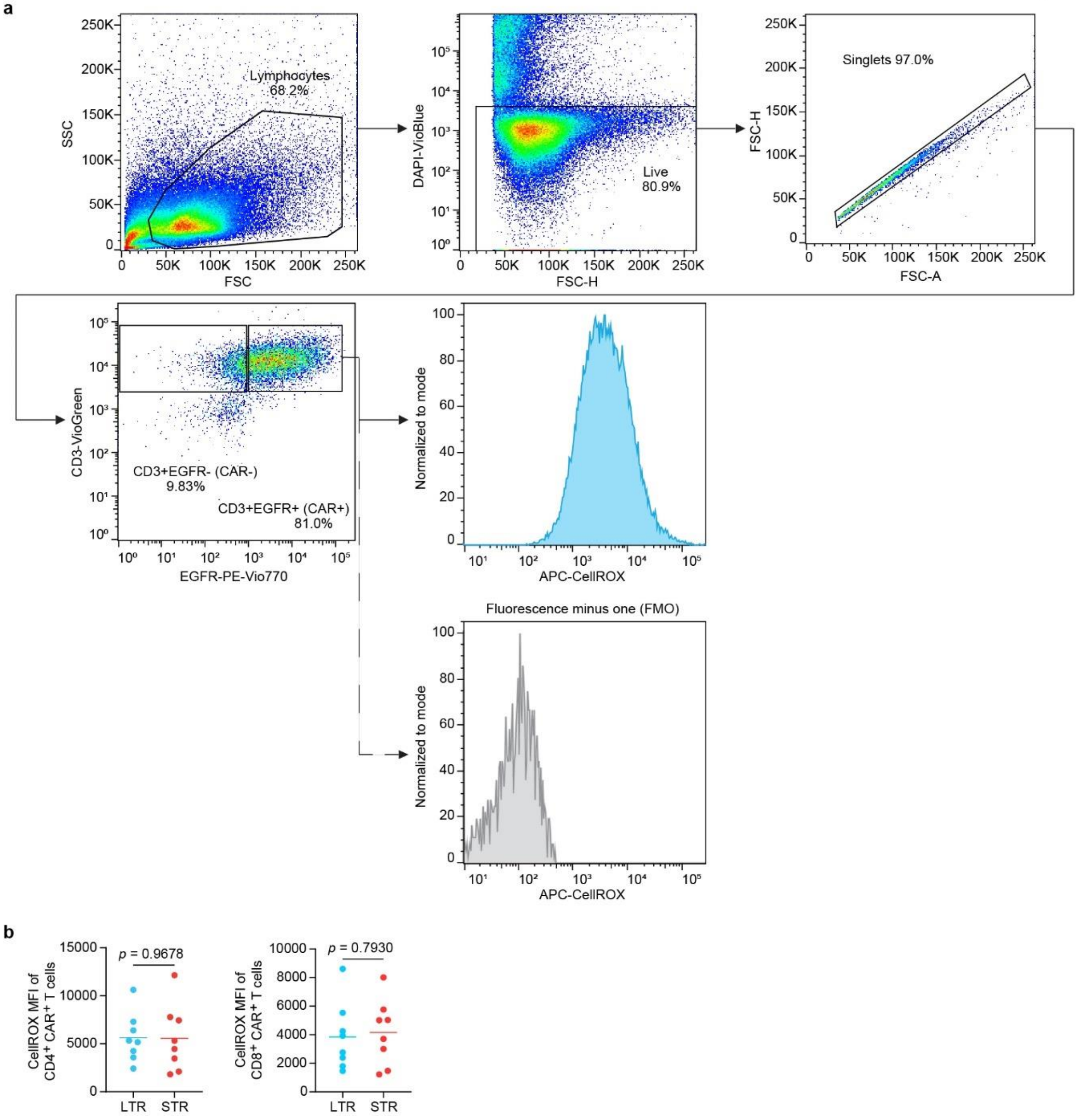

Supplementary Fig. 3: Reactive oxygen species assessment by flow cytometry. a Representative of gating strategy. All samples were FSC and SSC gated. DAPI negative cells were then gated, followed by FSC-A/FSC-H gating to select singlet cells. CAR T cells were detected using CD3 and EGFR staining, and reactive oxygen species level was assessed by measuring the CellROX fluorescent probe expression (%). **b** Analysis of CellROX expression in CD4+ and CD8+ CAR+ T cells products. Data are mean from $n = 16$ patients. Significance levels were calculated using two-tailed unpaired Student's t-tests. Source data are provided as a Source Data file.

Supplementary Fig. 4

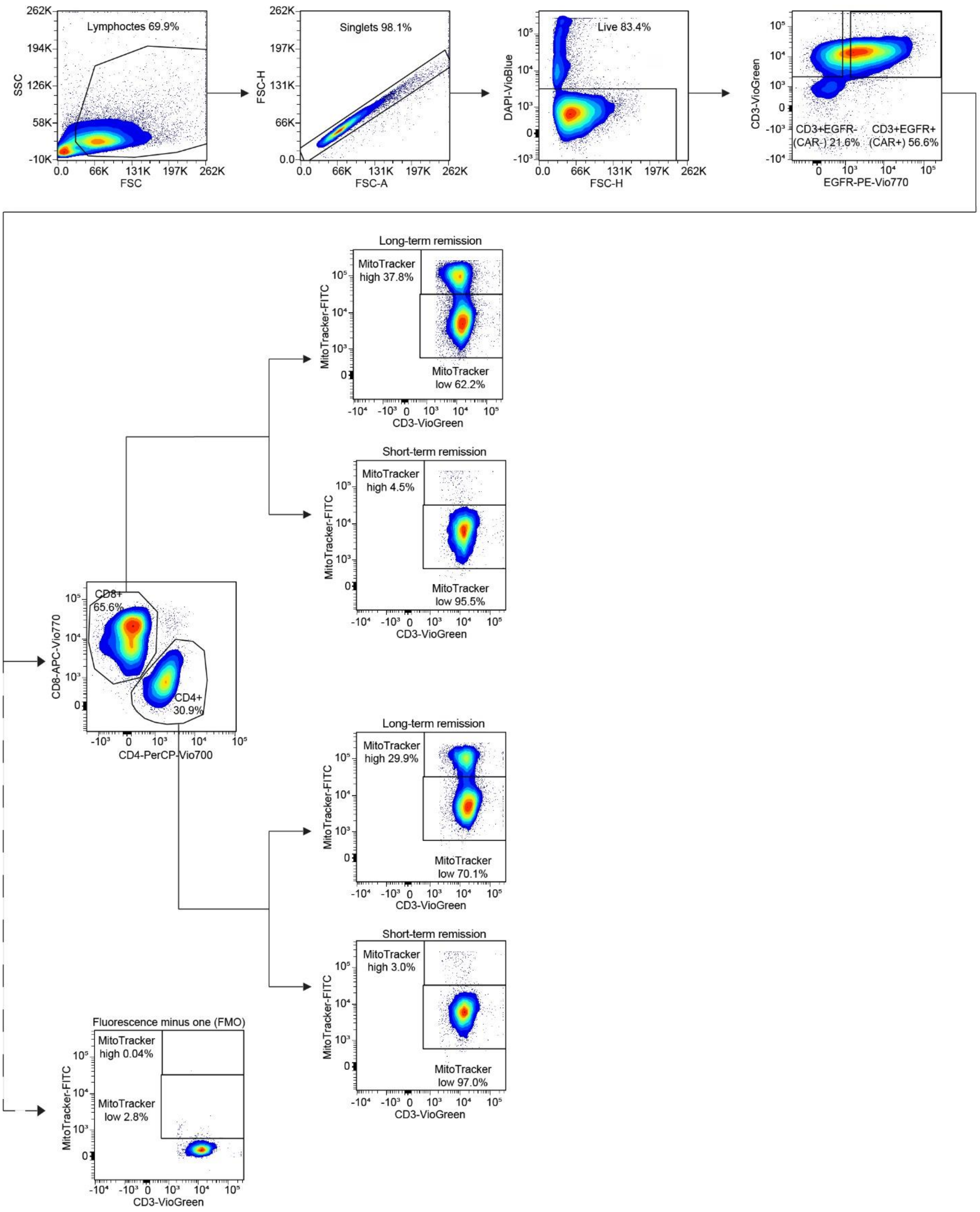

Supplementary Fig. 4: Mitochondrial mass assessment by flow cytometry. Representative of gating strategy. All samples were FSC and SSC gated, followed by FSC-A/FSC-H gating to select singlet cells. DAPI negative cells were then gated, and CAR and non-CAR T cells were detected using CD3 and EGFR staining. Mitochondrial mass was assessed by measuring the MitoTracker fluorescent probe expression (%). Corresponds to: Fig. 2b and Fig. 4q.

Supplementary Fig. 5

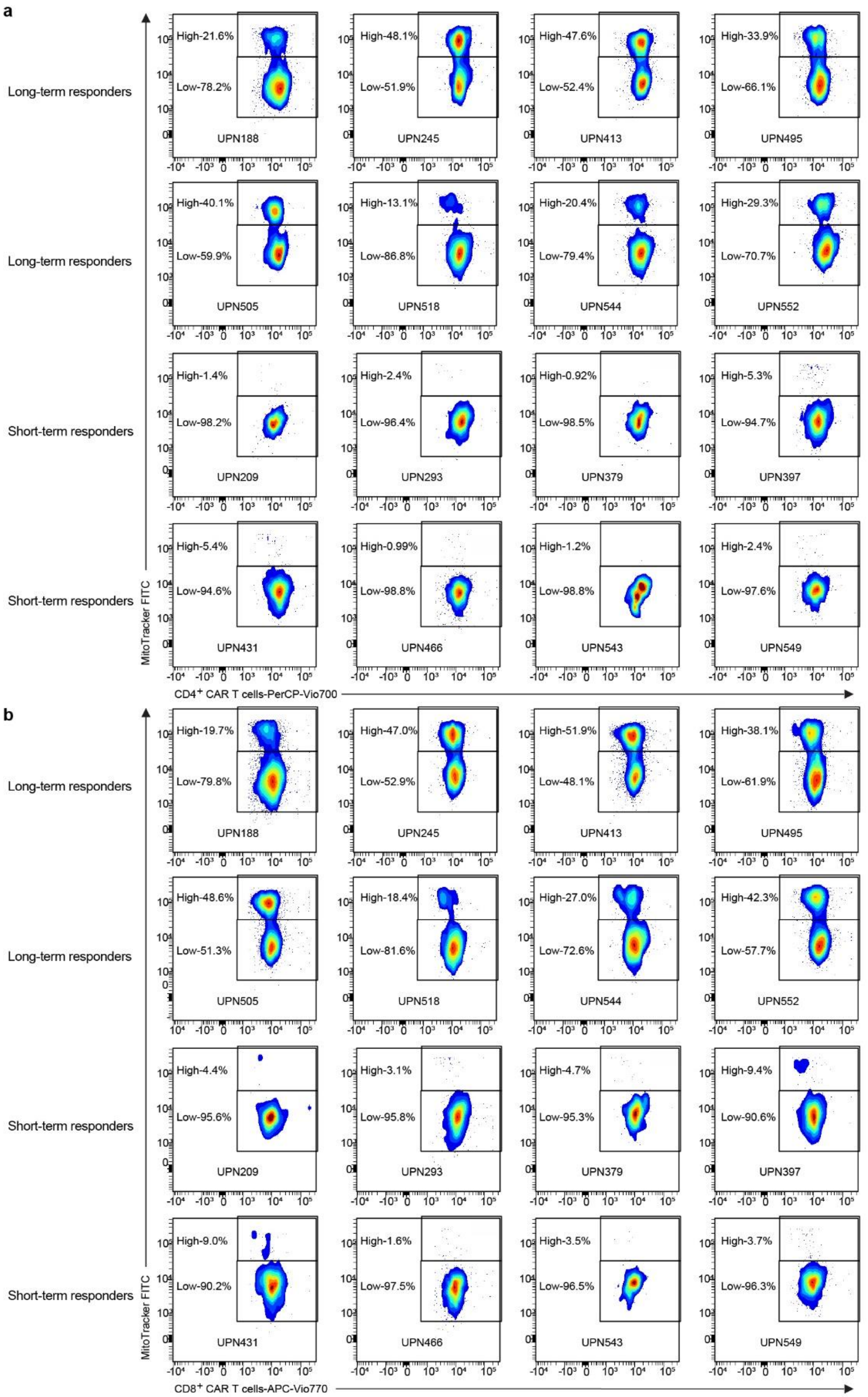

Supplementary Fig. 5: Mitochondrial mass assessment of pre-infusion CAR T cells in products of short- and long-term responders. Biaxial plots showing Mitotracker expression

level (%) in CD4+ (**a**) CD8+ **(b)** CAR+ T cells. *n* = 16 patients. Source data are provided as a Source Data file.

Supplementary Fig. 6

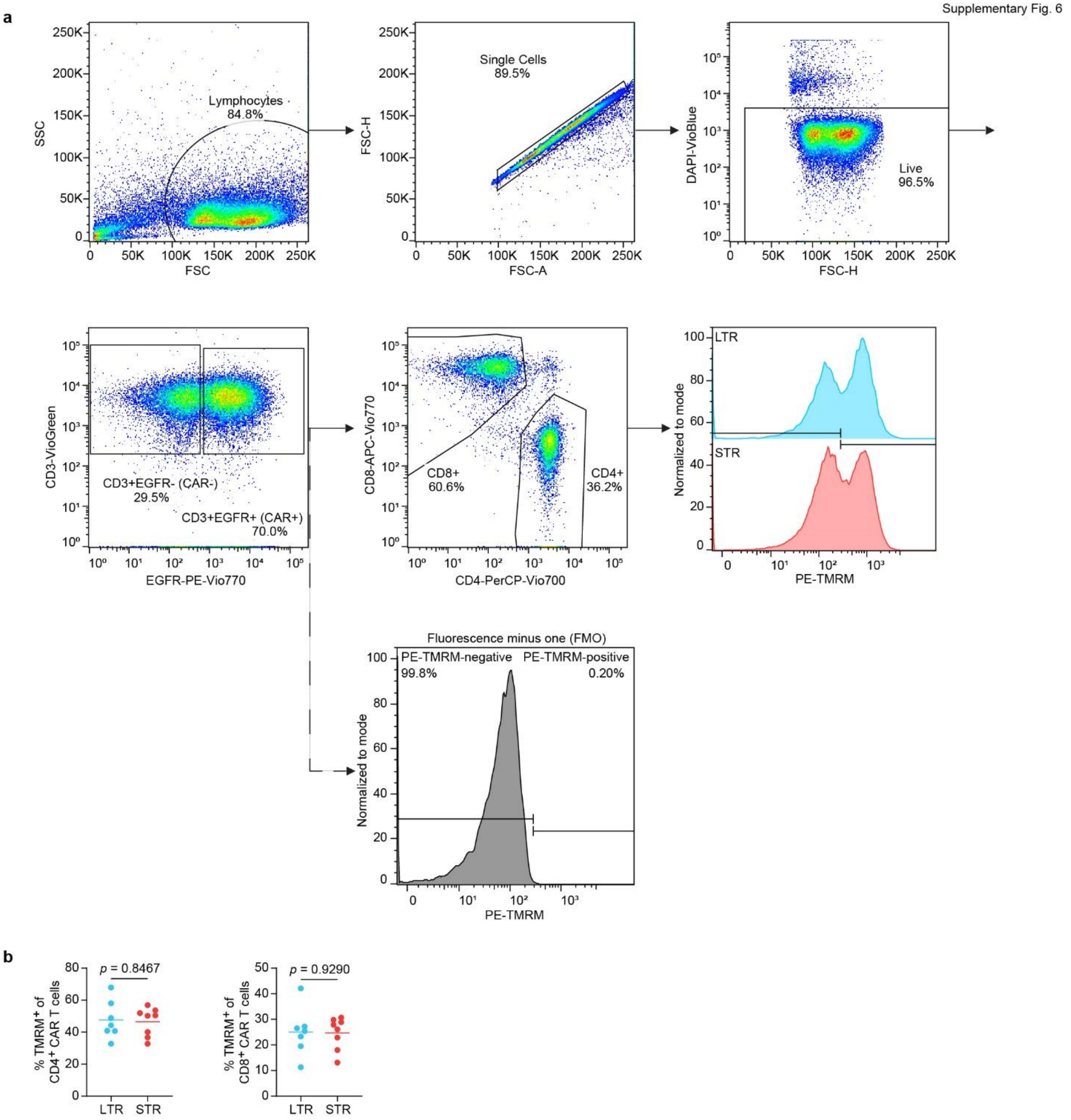

Supplementary Fig. 6: Mitochondrial membrane potential assessment by flow cytometry. **a** Representative of gating strategy. All samples were FSC and SSC gated, followed by FSC-A/FSC-H gating to select singlet cells. DAPI negative cells were then gated, and CAR cells were detected using CD3 and EGFR staining. Mitochondrial membrane potential was assessed by measuring the TMRM (Tetramethylrhodamine methyl ester) dye expression (%). **b** Analysis of TMRM expression in CD4+ and CD8+ CAR+ T cells pre-infusion products. Data are mean from *n*

= 6 biological replicates. Significance levels were calculated using two-tailed paired Student's *t*-tests. Source data are provided as a Source Data file.

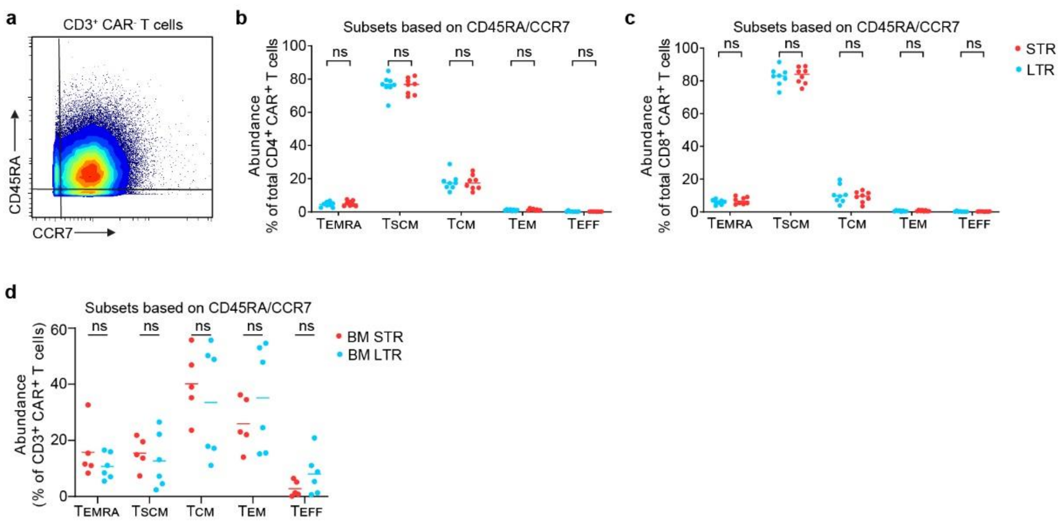

Supplementary Fig. 7: T cell differentiation subsets abundance by flow cytometry. a Representative biaxial plot to measure CAR T cell subsets using CD45RA and CCR7. Abundance of canonical T-cell subsets in CD4+ (**b**) and CD8+ (**c**) CAR+ T cells of pre-infusion products of short- and long-term responders. **d** Abundance of canonical T-cell subsets in CD3+ CAR+ T cells of day 28 post-infusion bone marrow samples of short- and long-term responders. Data are mean from $n = 16$ patients. ns, non-significant. Significance levels were calculated using two-tailed unpaired Student's *t*-tests. Source data are provided as a Source Data file.

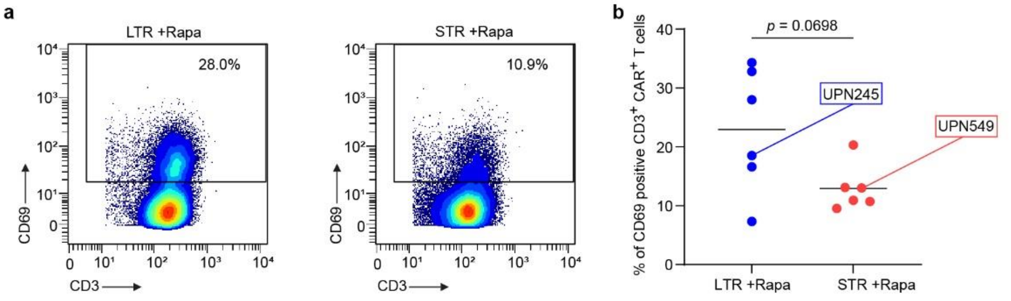

Supplementary Fig. 8: Comparison of CD69 expression in CAR T cell products of short- and long-term responders manufactured with Rapamycin. a Representative biaxial plots to measure CD69 expression in CD3+ CAR+ T cells. b CD69 expression in CD3+ CAR+ T cells products of short- and long-term responders manufactured with Rapamycin. Highlighted dots represent the CD69 expression level and UPN number in CAR T cell products used for the *in vivo* leukemia model experiment (**Fig. 5**). Data are mean from $n = 12$ patients. Significance levels were calculated using two-tailed unpaired Student's t-tests. Source data are provided as a Source Data file.
