# Supplementary material for: Immunometabolic determinants of long-term response in leukemia patients receiving CD19 CAR T cell therapy
Source: Nat Commun. 2026 Feb 20;17:2967. doi: 10.1038/s41467-026-69857-4 (PMC13035890; doi:10.1038/s41467-026-69857-4)
Supplement: Supplementary file 2 — Description of Additional Supplementary Files [file 41467_2026_69857_MOESM2_ESM.pdf]

Description of Additional Supplementary Files:

Supplementary Data 1: Detailed demographic and clinical characteristics of the patients. *n* = 16 patients.

Supplementary Data 2: A mass cytometry integrative panel.

Supplementary Data 3: CAR T cell metabolome dataset. *n* = 16 patients. Significance levels were calculated using two-tailed unpaired Student's *t*-tests.

Supplementary Data 4: Bone marrow serum metabolome dataset. *n* = 11 patients. Significance levels were calculated using two-tailed unpaired Student's *t*-tests.

Supplementary Data 5: CAR T cell rapamycin RNA dataset. Data are mean from *n* = 6 biological replicates. Significance levels were calculated using two-tailed paired Student's *t*-tests.

Supplementary Data 6: CAR T cell rapamycin metabolome dataset. Data are mean from *n* = 6 biological replicates. Significance levels were calculated using two-tailed paired Student's *t*-tests.
