# Supplementary material for: Immunometabolic determinants of long-term response in leukemia patients receiving CD19 CAR T cell therapy
Source: Nat Commun. 2026 Feb 20;17:2967. doi: 10.1038/s41467-026-69857-4 (PMC13035890; doi:10.1038/s41467-026-69857-4)
Supplement: Supplementary file 9 — Reporting Summary [file 41467_2026_69857_MOESM9_ESM.pdf]

nature portfolio

Corresponding author(s): Goldberg Lior

Last updated by author(s): Jan 8, 2026

Reporting Summary

Nature Portfolio wishes to improve the reproducibility of the work that we publish. This form provides structure for consistency and transparency in reporting. For further information on Nature Portfolio policies, see our Editorial Policies and the Editorial Policy Checklist.

Statistics

For all statistical analyses, confirm that the following items are present in the figure legend, table legend, main text, or Methods section.

n/a	Confirmed	
☐	☒	The exact sample size (*n*) for each experimental group/condition, given as a discrete number and unit of measurement
☐	☒	A statement on whether measurements were taken from distinct samples or whether the same sample was measured repeatedly
☐	☒	The statistical test(s) used AND whether they are one- or two-sided<br>*Only common tests should be described solely by name; describe more complex techniques in the Methods section.*
☒	☐	A description of all covariates tested
☒	☐	A description of any assumptions or corrections, such as tests of normality and adjustment for multiple comparisons
☐	☒	A full description of the statistical parameters including central tendency (e.g. means) or other basic estimates (e.g. regression coefficient) AND variation (e.g. standard deviation) or associated estimates of uncertainty (e.g. confidence intervals)
☐	☒	For null hypothesis testing, the test statistic (e.g. *F*, *t*, *r*) with confidence intervals, effect sizes, degrees of freedom and *P* value noted<br>*Give P values as exact values whenever suitable.*
☒	☐	For Bayesian analysis, information on the choice of priors and Markov chain Monte Carlo settings
☐	☒	For hierarchical and complex designs, identification of the appropriate level for tests and full reporting of outcomes
☒	☐	Estimates of effect sizes (e.g. Cohen's *d*, Pearson's *r*), indicating how they were calculated

Our web collection on statistics for biologists contains articles on many of the points above.

Software and code

Policy information about availability of computer code

Data collection	No software was used for data collection.
Data analysis	Cytokine concentrations were calculated using Bio-Plex Manager 6.2 software.<br>All statistical comparisons were made in GraphPad Prism 10.2.1.

For manuscripts utilizing custom algorithms or software that are central to the research but not yet described in published literature, software must be made available to editors and reviewers. We strongly encourage code deposition in a community repository (e.g. GitHub). See the Nature Portfolio guidelines for submitting code & software for further information.

Data

Policy information about availability of data

All manuscripts must include a data availability statement. This statement should provide the following information, where applicable:

- Accession codes, unique identifiers, or web links for publicly available datasets
- A description of any restrictions on data availability
- For clinical datasets or third party data, please ensure that the statement adheres to our policy

The RNA sequencing data generated in this study are available at the Gene Expression Omnibus (GEO) repository of the National Center for Biotechnology Information under accession code GSE298663. Metabolomic profiles are available at the NIH Common Fund's National Metabolomics Data Repository (NMDR) website, the Metabolomics Workbench42 (https://www.metabolomicsworkbench.org, Study IDs: ST003963, ST003964, ST003966. Supplementary information, including Supplementary Figs. 1–8 and Supplementary Data 1–6 are provided with the online version of this paper. All other datasets generated during and/or

analyzed during this study are available from the corresponding author on request. Source data are provided with this paper.

Research involving human participants, their data, or biological material

Policy information about studies with human participants or human data. See also policy information about sex, gender (identity/presentation), and sexual orientation and race, ethnicity and racism.

Reporting on sex and gender	Patient's sex was self-reported and recorded in Table 1. Sex-based statistical analysis was not performed due to our small sample size.
Reporting on race, ethnicity, or other socially relevant groupings	Race, ethnicity, or other socially relevant grouping were not applicable to this retrospective cohort study.
Population characteristics	See table 1 and supplementary table 1 in the included submission for relevant baseline and on-treatment patient characteristics.
Recruitment	Participants were recruited in a prospective institutional biobanking protocol which collects clinical data along with biospecimens for future research. All patients undergoing CAR T-cell therapy were eligible for participation in this biobanking study.
Ethics oversight	The study was conducted in accordance with the Declaration of Helsinki and with the approval of the City of Hope Internal Review Board (IRB #13447).

Note that full information on the approval of the study protocol must also be provided in the manuscript.

Field-specific reporting

Please select the one below that is the best fit for your research. If you are not sure, read the appropriate sections before making your selection.

[x] Life sciences   [ ] Behavioural & social sciences   [ ] Ecological, evolutionary & environmental sciences

For a reference copy of the document with all sections, see nature.com/documents/nr-reporting-summary-flat.pdf

Life sciences study design

All studies must disclose on these points even when the disclosure is negative.

Sample size	Analysis was performed based on longitudinal CAR T-cell data from 16 patients. Sample size was chosen based on availability of patients/samples.
Data exclusions	No data was excluded.
Replication	Findings were consistent across all patients.
Randomization	To study the immunometabolic characteristics associated with response to CAR T cell therapy, we investigated the pre-infusion CAR T cell products and biological samples from a cohort of sixteen patients treated in our clinical trial, all of whom responded to CD19-CAR T cell therapy. The cohort included two sub-groups (n=8 per sub-group) based on length of response to CAR T cells, with short-term responders (STR) having CD19+ leukemia relapse within 142±30 days following initial response and long-term responders (LTR) maintaining remission without subsequent therapy post-CAR T cell infusion. We pairwise matched the sub-groups, and both cohorts had similar demographic and clinical characteristics other than length of response, allowing us to study CAR T cell functionality without potential confounding variables such as antigen escape and post CAR T cell consolidation therapies (Table 1, Supplementary Data 1).
Blinding	There was no blinding in this CAR T cell trial.

Reporting for specific materials, systems and methods

We require information from authors about some types of materials, experimental systems and methods used in many studies. Here, indicate whether each material, system or method listed is relevant to your study. If you are not sure if a list item applies to your research, read the appropriate section before selecting a response.

Materials & experimental systems

n/a	Involved in the study
☐	☒ Antibodies
☐	☒ Eukaryotic cell lines
☒	☐ Palaeontology and archaeology
☐	☒ Animals and other organisms
☐	☒ Clinical data
☒	☐ Dual use research of concern
☒	☐ Plants

Methods

n/a	Involved in the study
☒	☐ ChIP-seq
☐	☒ Flow cytometry
☒	☐ MRI-based neuroimaging

Antibodies

Antibodies used

106Cd CD45 Fluidigm 3106001B HI30
141Pr CD3 Fluidigm 3154010B UCHT1
143Nd CD45RA Fluidigm 3153004B HI100
144Nd CD69 Fluidigm 3143006B FN50
145Nd CD4 Fluidigm 3175008B RPA-T4
146Nd CD8a Fluidigm 3149010B RPA-T8
147Sm Lactate Dehydrogenase A (LDHA) Fluidigm 3167009A 44/ALDH
148Nd AMP-activated protein kinase (AMPK) Abcam ab210714 Y365
149Sm CD25 (IL-2Ra) Fluidigm 3156029B 2A3
150Nd Glucose Transporter Protein Type 1 (GLUT1) abcam ab115730 EPR3915
151Eu Stearoyl-CoA Desaturase 1 (SCD1) abcam ab19862 CD.E10
152Sm pAkt [S473] Fluidigm 3152005A D9E
153Eu ATP5A Abcam ab14748 15H4C4
154Sm T-Cell Immunoglobulin and Mucin Domain 3 (TIM-3) Fluidigm 3174018B F38-2E2
155Gd CD36 Fluidigm 3158010B 5-271
158Gd CD27 Fluidigm 3144018B L128
159Tb CD98 Fluidigm 3141019B UM7F8
160Gd Lysosomal Acid Lipase (LAL) abcam ab36597 9G7F12
161Dy Ki-67 Fluidigm 3161007B B56
162Dy Carnitine Palmitoyl Transferase 1A (CPT1A) abcam ab128568 8F6AE9
163Dy ATP Citrate Lyase (ACLY) abcam ab227996 EP704Y
164Dy Glucose-6-Phosphate Dehydrogenase (G6PD) abcam ab210702 EPR20668
165Ho CD45RO Fluidigm 3165011B UCHL1
166Er Fatty acid synthase (FASN) abcam ab128870 EPR7466
167Er CC Chemokine Receptor Type 7 (CCR7) Fluidigm 3167009B G043H7
168Er Citrate Synthase (CS) abcam ab129095 EPR8067
169Tm Hexokinase (HK) abcam ab104836 3D3
170Er EGFR Fluidigm 3173005B AY13
171Yb Glyceraldehyde-3-Phosphate Dehydrogenase (GAPDH) Santa Cruz Biotechnology AM4300 6C5
172Yb pS6 [S235/S236] Fluidigm 3145001B N7-548
173Yb Granzyme B Fluidigm 3173006B GB11
174Yb Acyl-CoA Dehydrogenase Medium Chain (ACADM) abcam ab110296 3B7BH7
175Lu CD279 (PD-1) Fluidigm 3175008B EH12.2H7
164Dy CyclinB1 Fluidigm 3164010A GNS-1
166Er pRb Fluidigm 3166011A J112-906
176Yb Mammalian Target of Rapamycin (mTOR) Cell Signaling Technology 47102 7C10
194Pt CD45 Fluidigm 3194001B HI30
198Pt CD45 Fluidigm 3198001B HI30
209Bi TIGIT Fluidigm 3209013B MBSA43
89Y CD45 Fluidigm 3089003B HI30
Brilliant Violet 510 CD3 BD Bioscience 563109 UCHT1
PE CD3 BD Bioscience 347347 SK7
PerCP CD4 BD Bioscience 347324 SK3
APC-C7 CD8 BD Bioscience 348793 SK1
PE EGFR BD Bioscience 352906 EGFR.1

Validation

All antibodies were validated by the manufacturer for reactivity to human antigens. All antibodies were either validated or recommended for flow cytometry. Validation statements of all antibodies used in this manuscript were noted on the manufacturer's website with relevant citations.

Eukaryotic cell lines

Policy information about cell lines and Sex and Gender in Research

Cell line source(s)	Nalm 6 cell line was obtained from the ATCC. 018z cell ine was obtianed from Prof. Shai Izraeli Lab.
Authentication	Short tandem repeat cell line authentication was performed by ATCC/Prof. Shai Izraeli Lab prior to delivery.
Mycoplasma contamination	Cell lines were tested once every six months for general use. Before using for in vivo experiments, cell lines are tested with MycoAlert detection kit (Lonza).
Commonly misidentified lines (See ICLAC register)	n/a

Animals and other research organisms

Policy information about studies involving animals; ARRIVE guidelines recommended for reporting animal research, and Sex and Gender in Research

Laboratory animals	6-8 week old male or female NOD/SCID/IL2Rg (NSG) mice were used for all in vivo experiments.
Wild animals	The study did not involve wild animals.
Reporting on sex	Sex of animals was not considered in study design. All animals were sex and age matched in each experiment. The authors conclude that the findings are applicable, relevant, and reproducible for both males and females based on observed findings.
Field-collected samples	No field collected samples were used in this study.
Ethics oversight	Animal Resource Center of City of Hope in accordance with the approved Institutional Animal Care and Use Committee guidelines (IACUC: 21034).

Note that full information on the approval of the study protocol must also be provided in the manuscript.

Clinical data

Policy information about clinical studies

All manuscripts should comply with the ICMJE guidelines for publication of clinical research and a completed CONSORT checklist must be included with all submissions.

Clinical trial registration	NCT02146924.
Study protocol	Study protocol can be accessed at ClinicalTrails.gov. https://clinicaltrials.gov/study/NCT02146924.
Data collection	Clinical data was collected from the clinical electronic medical record at City of Hope.
Outcomes	The cohort included two sub-groups (n=8 per sub-group) based on length of response to CAR T cells, with short-term responders (STR) having CD19+ leukemia relapse within 142±30 days following initial response and long-term responders (LTR) maintaining remission without subsequent therapy post-CAR T cell infusion.

Plants

Seed stocks	n/a
Novel plant genotypes	n/a
Authentication	n/a

Flow Cytometry

Plots

Confirm that:

- [x] The axis labels state the marker and fluorochrome used (e.g. CD4-FITC).
- [x] The axis scales are clearly visible. Include numbers along axes only for bottom left plot of group (a 'group' is an analysis of identical markers).
- [x] All plots are contour plots with outliers or pseudocolor plots.
- [x] A numerical value for number of cells or percentage (with statistics) is provided.

Methodology

Sample preparation	For surface staining, cells were incubated with fluorochrome-conjugated monoclonal antibodies. Cells were resuspended in FACS buffer, which consists of HBSS (Gibco, 14175095), 2% FBS and NaN3 (Sigma, S8032), and incubated with antibodies at 4°C in the dark. Following a washing step with FACS buffer and surface antibody staining, DAPI (Invitrogen, D21490) was added for viability staining before analysis.
Instrument	MACSQuant Analyzer 10 (Miltenyi Biotech, 130-096-343)
Software	FlowJo
Cell population abundance	CAR T cell abundance ranged from 50-90% of total live CD3 positive cells.
Gating strategy	All samples are gated on FSC/SSC lymphocyte populations, single cells (Using FSC-W/FSC-H and SSC-W/SSC-H), live populations, then CD3+, CAR+ and CD4+/CD8+. Frequency of positive gates is determined using an isotype or FMO control.

- [x] Tick this box to confirm that a figure exemplifying the gating strategy is provided in the Supplementary Information.
